# Side effects of chaperone gene co-expression in recombinant protein production

**DOI:** 10.1186/1475-2859-9-64

**Published:** 2010-09-02

**Authors:** Mónica Martínez-Alonso, Elena García-Fruitós, Neus Ferrer-Miralles, Ursula Rinas, Antonio Villaverde

**Affiliations:** 1 Institute for Biotechnology and Biomedicine, Universitat Autònoma de Barcelona, Bellaterra, 08193 Barcelona, Spain; 2 Department of Genetics and Microbiology, Universitat Autònoma de Barcelona,08193, Bellaterra Barcelona, Spain; 3 CIBER de Bioingeniería, Biomateriales y Nanomedicina (CIBER-BBN), Bellaterra, 08193 Barcelona, Spain; 4 Helmholtz Centre for Infection Research, Inhoffenstr. 7, 38124 Braunschweig, Germany; 5 Leibniz University Hannover, Life Science - Technical Chemistry, Callinstr. 5, 30167 Hannover, Germany

## Abstract

Insufficient availability of molecular chaperones is observed as a major bottleneck for proper protein folding in recombinant protein production. Therefore, co-production of selected sets of cell chaperones along with foreign polypeptides is a common approach to increase the yield of properly folded, recombinant proteins in bacterial cell factories. However, unbalanced amounts of folding modulators handling folding-reluctant protein species might instead trigger undesired proteolytic activities, detrimental regarding recombinant protein stability, quality and yield. This minireview summarizes the most recent observations of chaperone-linked negative side effects, mostly focusing on DnaK and GroEL sets, when using these proteins as folding assistant agents. These events are discussed in the context of the complexity of the cell quality network and the consequent intricacy of the physiological responses triggered by protein misfolding.

## Review

Poor product quality is a common event in the biological synthesis of target proteins and a major cause for recombinant enzymes and pharmaceuticals to be excluded from the market [1]. Recombinant protein misfolding and the triggering of the consequent cell responses are both general events among microbial cell factories [2]. Although what protein quality means might be highly controversial [3-5], it is in general assumed that the soluble protein version, despite the potential occurrence of soluble aggregates [4,6-10] and the presence of functional protein species in protein aggregates [4,11-15], is the most desirable form of the final product of a protein production process. Traditionally, gaining solubility has been approached by tuning-down the production rate (e.g. by decreasing temperature), reducing recombinant gene dosage or the strength of the promoter, or supplying additional amounts of host chaperones, as they are seen as limiting during the overproduction of misfolding-prone protein species [16,17].

Under the high substrate load context of recombinant cells, chaperones, main players in the quality control system, might be over-titrated and therefore their protein targets excluded from folding pathways leading to the native conformation, accumulating as refractile particles called inclusion bodies (IBs) [13,18]. Therefore, several individual chaperones or chaperone sets have been selected for overproduction along with the target recombinant protein. In *Escherichia coli (E. coli)*, most of these approaches have involved the two main cytosolic chaperones, namely DnaK and GroEL, as well as some of their co-chaperones [6,19]. However, the fine examination of physiological responses to protein production in bacteria and other microorganisms [2,10,20-22], has revealed that chaperone co-production, as a quality-addressed strategy, might eventually show undesirable side effects regarding protein yield and quality (Table 1). Here we summarize the main indications pointing out the chaperone side-effects, mainly focusing on DnaK, GroEL and their cooperating folding modulators.

**Table 1:** Main undesired side effects observed during chaperone co-production on the quality and yield of recombinant proteins produced in *E. coli*, as exemplified by representative studies.

Chaperone/Chaperone set	Recombinant protein	Effects on protein production	References
DnaKJE	Horseradish peroxidase	Growth inhibition	[31]
DnaKJ	Aggregation-prone GFP	Proteolyis, reduced yield and lower conformational quality	[20,21]
DnaKJE and/or Trigger Factor	Guinea pig liver transglutaminase	Reduced specific activity	[74]
DnaKJE, ClpB and GroELS	Basic fibroblast growth factor	Reduced yield	[22]
DnaKJE-GroELS-ClpB and Trigger Factor	Human protein kinase catalytic domains	Increased soluble aggregate formation	[43]
GroELS	Basic fibroblast growth factor	Proteolysis, reduced yield	[22]
Trigger Factor and GroELS	N-acyl-D-amino acid amidohydrolases	Reduced specific activity	[75]
GroELS	Fab Antibody Fragment	Reduced yield	[46]
GroELS	scFv antibody fragment	Reduced solubility	[76]
GroELS	Cyclodextrin glycosyltransferase	Reduced specific activity	[32]

## DnaK

DnaK, homolog of the eukaryotic Hsp70, is the major cytosolic chaperone in *E. coli*, and plays an important role in the control of conformational quality. In fact, DnaK is involved in different activities such as prevention of aggregation, folding and refolding of misfolded species and protein disaggregation [23-27]. For this reason, DnaK has often been used in co-production approaches, either together with its co-chaperone DnaJ [20,28] or both with DnaJ and their nucleotide exchange factor GrpE [6,29-36] to minimize aggregation and to enhance solubility of the recombinant protein [37-40]. Co-chaperones have been observed as necessary because over-expression of *dnaK *gene alone is toxic for the cell, leading to growth inhibition and abnormal septation [41]. Although this approach has proven to be useful in many cases [34,42], this set of folding modulators has not been consistently successful to enhance solubility of target recombinant proteins [43-45] and solubility has been often enhanced at expenses of protein yield [29,32,34]. This fact has been attributed both to cell growth inhibition [31,35,46] and to the proteolysis of the recombinant protein [20,31,35]. Besides its mentioned activities, recent publications describe that DnaK is also involved in the degradation of aggregation-prone but functional (or suitable to be activated) polypeptides by targeting them to proteases such as Lon and ClpP [47-49]. In fact, absence of a functional DnaK results in increased proteolytic resistance of a target protein, the half-life of which is increased up to three-fold in these conditions [21]. Hence, this dual role of the chaperone, which acts both as a folding modulator and as proteolytic enhancer, contributes to explain the divergence of results obtained upon its co-production. Although recombinant protein solubility can be improved by high levels of DnaK and its co-chaperones, protein quality might be compromised since an important part of this effect is obtained by increasing soluble aggregate species [9,21] with variable specific activity. In addition, yield of recombinant protein decreases due to the proteolysis stimulation carried out by DnaK [22]. The occurrence of such a DnaK-mediated side effect proves that strategies developed to optimize recombinant protein production processes have to be redefined, considering that solubility and conformational quality are independently controlled.

Moreover, because DnaK is also a negative regulator of the heat shock response [50], an enhanced concentration of DnaK above physiological levels can result in down-regulation of other heat shock proteins. Actually, decreased levels of GroEL chaperone have been reported in DnaK-overproducing cells [28,51]. Thus, taking into account that selection of the appropriate set of folding modulators is still a trial and error process, this scenario may then result in a more pronounced folding impairment for proteins that not only require interaction with DnaK but also with the GroEL system.

## GroEL(S)

The GroELS heat shock chaperone team is of vital importance for *E. coli *with GroEL being an essential protein for growth at all temperatures [52]. Co-production of this chaperone team has been widely applied to improve the solubility of proteins which tend to form IBs, in many cases with remarkable success [37,38,53,54]. However, also failures of GroELS to improve solubility have been reported, mostly the impact of GroELS was neutral, namely without increasing the amount of properly folded protein [55,56] or decreasing the amount of IB-deposited target protein [57]. In particular, failures of GroELS co-production for improved target protein solubility have been observed when aiming for production of large proteins [55]. This is a comprehensible finding as large proteins can not enter the cavity formed by the GroEL chaperone [58] thus leading to a preference of GroEL for substrate proteins in the molecular mass range of 10/20 - 55/60 kDa [59-61].

In addition, past studies also indicated that GroEL is involved in promoting proteolytic degradation through target protein binding [62-65]. In fact, the natural role of GroEL not only includes chaperoning functions but also encompasses a vital role in fostering proteolytic degradation. For example, GroEL plays a central role in promoting proteolytic degradation of a regulatory protein to reduce potentially detrimental effects of non-tuned gene expression [66]. In addition, GroELS is also involved in "protein trash removal", namely fostering proteolytic degradation of endogenous protein aggregates generated during heat shock [67].

A detailed study on the involvement of GroELS in target protein degradation was carried out during temperature-induced production of basic fibroblast growth factor [22]. Temperature-induced production leads to the formation of soluble growth factor and growth factor deposited in the form of IBs [68]. Protein purified from the soluble cell fraction of temperature-induced cells is biologically active as determined by mitogenic activity measurements [69]. Co-production of GroELS does not prevent IB formation but leads to complete IB dissolution followed by proteolytic degradation of basic fibroblast growth factor [22]. In this case, IB dissolution followed by proteolytic degradation of the target protein was more efficient with GroELS than with the DnaKJ/GrpE system.

### Solving chaperone-promoted proteolysis

Despite the mentioned reports indicating DnaK-induced proteolysis upon recombinant protein production, it is difficult to find in the literature any attempt to solve this problem. Even in *E. coli *genetic backgrounds knockout for the main cytosolic protease gene (Lon), proteolytic activity is still a hurdle to recombinant protein production probably by induction of other proteolytic systems [70]. However, in a recent study [71] we addressed this issue by re-hosting DnaK and its co-chaperone DnaJ into a system lacking orthologs of the bacterial proteases responsible for the protein degradation mediated by DnaK. The goal of such approach was to uncouple the valuable folding activity of DnaK from its other activities linked to proteolysis. Because DnaK has been highly conserved in evolution (DnaK homologs can be found in all kingdoms of life) the reasoning was that its folding activity could be conserved in other organisms, but not so the associated proteolytic activity because it is dependent on the bacterial proteases Lon and ClpP. Insect cells were chosen as the host for the *E. coli *DnaKJ chaperone pair, which was introduced in the production system upon infection of the cells with recombinant baculovirus vectors carrying the corresponding genes. In this eukaryotic system, chaperone gene co-expression resulted in enhanced yield and biological activity of a reporter protein, which also showed increased stability in presence of the bacterial chaperones, indicative of absence of DnaK-mediated proteolysis. This was in marked contrast to what had previously been described in *E. coli *for the production of the same protein and chaperone combination [21]. The same study also showed positive effects of the set of bacterial folding modulators on the production of three other recombinant proteins in the insect cell-baculovirus system, namely VP1 and VP2 from the capsid of Foot-and-Mouth Disease Virus, and human α-galactosidase. A later, related study [72] extended this approach to an *in vivo *model by using the recombinant baculoviruses encoding the bacterial chaperones to infect insect larvae, a system of use as a biofactory but where yields are usually reduced due to protein aggregation. In this system, absence of DnaK-induced proteolysis was also evident, and co-production of the bacterial chaperones boosted protein solubility by almost 100%. Taken together, these studies not only show how the effective discrimination of activities has been a suitable strategy to exclude the undesirable effects of the DnaKJ chaperone pair, but also prove that bacterial folding modulators are functional in other recombinant systems.

## Conclusions

**Figure 1:**
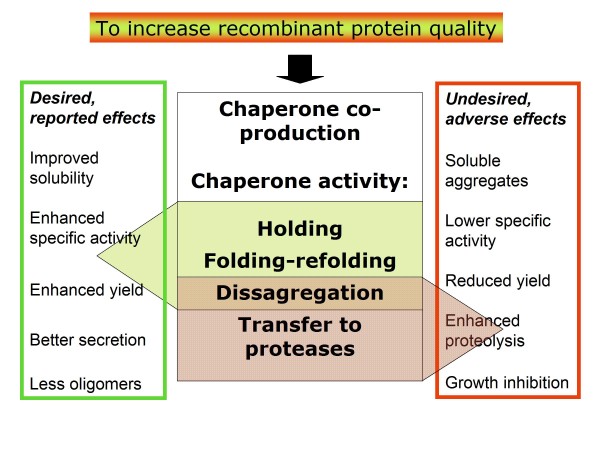
**Aimed to increase recombinant quality and solubility, co-production of individual chaperones or chaperone sets has been a common strategy since the role of these proteins in quality control has been solved, mainly involving protein holding to prevent aggregation, folding or refolding activities and disaggregation from inclusion bodies**. Many studies report on the positive effects of chaperone gene co-expression, regarding solubility, yield, secretion ability and specific activity (green box). However, it is also true that this strategy has been largely controversial and the eventual success seen as highly product- and/or process-dependent. Also, more recent studies reveal that an excess of certain chaperones has negative effects on protein yield and other parameters related to protein quality (red box), mainly due to the role of chaperones in promoting proteolysis of folding reluctant proteins. This promotion of proteolysis seems to be mechanistically linked to the disaggregation activities ruled by DnaK [21,77].

Despite their proven success as folding modulators in protein production processes, bacterial chaperones (mainly DnaK and GroEL and associated cofactors) also show undesired side effects related to their activities in promoting proteolysis of target proteins (Figure 1). This fact might account, at least partially, for the inconsistent results reported upon the use of these chaperones in years of exploitation of microbial cell factories for protein production. Because of the lack of coincidence and the divergent control of protein solubility and quality observed in bacteria [3,21], chaperone co-production might have enhanced solubility as a consequence of an undesired reduction of recombinant protein yield. Probably, most failures of chaperone gene co-expression on target protein solubility have not been reported in the scientific literature (including our own observations) and, in some cases, a supposed positive effect of chaperone co-production might just reflect the presence of soluble aggregates but not of functional protein [43]. Moreover, over-production of chaperones as over-production of any other protein can contribute to the metabolic burden thereby leading to growth rate reduction as well as decreased final biomass yields [73]. As a first example, re-hosting of bacterial chaperones has proven to be a way to disconnect folding assistance and proteolysis. However, further studies are still needed to explore other alternative ways to systematically minimize chaperone side effects in protein production, keeping their desired activities on folding-reluctant recombinant proteins.

## Competing interests

The authors declare that they have no competing interests.

## Authors' contributions

All authors have contributed to this review from their complementing areas of expertise and have read and approved the final manuscript.
